# A tradeoff between bacteriophage resistance and bacterial motility is mediated by the Rcs phosphorelay in Escherichia coli

**DOI:** 10.1099/mic.0.001491

**Published:** 2024-08-28

**Authors:** Alita R. Burmeister, Harleen Tewatia, Chloé Skinner

**Affiliations:** 1 Department of Biological Sciences, University of Wisconsin Milwaukee, Milwaukee, WI, USA

**Keywords:** all-or-nothing pleiotropy, bacteria, bacteriophage, continuous pleiotropy, phosphorelay, resistance

## Abstract

Across the tree of life, pleiotropy is thought to constrain adaptation through evolutionary tradeoffs. However, few examples of pleiotropy exist that are well explained at the genetic level, especially for pleiotropy that is mediated by multiple genes. Here, we describe a set of pleiotropic mutations that mediate two key fitness components in bacteria: parasite resistance and motility. We subjected *Escherichia coli* to strong selection by phage U136B to obtain 27 independent mucoid mutants. Mucoidy is a phenotype that results from excess exopolysaccharide and can act as a barrier against viral infection but can also interfere with other cellular functions. We quantified the mutants’ phage resistance using efficiency of plaquing assays and swimming motility using swim agar plates, and we sequenced the complete genomes of all mutants to identify mucoid-causing mutations. Increased phage resistance co-occurred with decreased motility. This relationship was mediated by highly parallel (27/27) mutations to the Rcs phosphorelay pathway, which senses membrane stress to regulate exopolysaccharide production. Together, these results provide an empirical example of a pleiotropic relationship between two traits with intermediate genetic complexity.

## Introduction

Pleiotropy – which occurs when a genetic change results in alteration of two or more phenotypes – is of importance throughout both fundamental and applied biology. In evolutionary biology, pleiotropy can result in tradeoffs when it has fitness consequences in two or more environments. These consequences can constrain evolution and contribute to the maintenance of diversity. For example, the evolution of parasite resistance can occur at a cost to other fitness components, resulting in the coexistence of susceptible and resistant hosts [1]. Pleiotropy is also increasingly being leveraged to understand pathogen evolution and create effective treatments. In particular, bacteriophage-based alternatives to antibiotics can be more effective when phage resistance comes at a cost to bacterial virulence [2,4]. In these cases, specific phages could be applied to not only kill their target host bacteria, but to also evolutionarily ‘steer’ bacterial populations towards a less pathogenic state via pleiotropic mutations [3].

Prior work has sought to characterize the causes and effects of pleiotropy. This has included the identification of genes underlying multiple measurable traits (‘pleiotropic’ genes). Identification of pleiotropic genes is often complicated by genetic linkage or by functional redundancy, where more than one gene may impact the same phenotypes [5]. For example, in *Mimulus guttatus*, at least 24 genetic polymorphisms contribute to antagonistic pleiotropy between flower size and days to flower [6]. Because of these complications, it can be difficult to generate predictions about how specific mutations may impact other traits, even when one or only a few loci are involved. Some single-gene loci have been identified that underly multiple measurable traits that have fitness effects and therefore consequences for evolution. For example, in humans the haemoglobin S gene can confer partial malaria resistance but comes with the pleiotropic effect of sickle-cell anaemia [7]. In bacteria, examples of single-gene pleiotropy occur when a gene encodes a protein that is exploited by a bacteriophage as a receptor. In those cases, mutational loss or modification of the gene can lead to phage resistance while conferring the loss of other fitness components, such as sugar transport or infectivity [2,4,8]. However, little work has characterized pleiotropy caused by mutations in a set of several genes.

We previously characterized a candidate therapeutic phage (U136B) for its potential to leverage single-gene pleiotropy to steer antibiotic resistance phenotypes of bacterial populations. Phage U136B infects *Escherichia coli* through the outer membrane protein TolC [9], an antibiotic efflux pump component [10]. When bacterial populations evolved resistance to phage U136B, they often had mutations that severely altered the TolC structure. These mutations frequently resulted in reduced antibiotic resistance [9]. We also identified mutations that modified the lipopolysaccharide (LPS) structure, pleiotropically decreasing resistance to one antibiotic and increasing resistance to another, probably through changes to the cell’s antibiotic permeability [9]. Therefore, pleiotropy depended not only on the specific phage resistance mutation but also on the specific antibiotic tested, highlighting the need to understand potential roadblocks and alternative routes to phage resistance.

One such alternative route to phage resistance is the multi-gene-encoded ‘mucoid’ phenotype (mucoidy) [11,12]. Mucoid bacteria produce large, viscous colonies, resulting from excess polysaccharide or other macromolecules coating the outside of the cell [11]. This coating is thought to be a general resistance mechanism to phages [12] by limiting phage access to the cell surface and blocking phage receptor-specific adsorption, such as for phages T7 and lambda [13]. By limiting access to the cell surface receptors, the mucoidy phenotype also provides resistance to type VI secretion system-mediated attack from competitive bacteria [14]. In contrast, for bacteria that are naturally mucoid, some phages target the mucoid capsule during host recognition [15,17]. In that case, non-mucoid or mucoid variant mutations may confer phage resistance [18].

The mucoidy phenotype can be caused by mutations in a variety of genes and genetic pathways in *E. coli* [19,21] and is thus more complex than the single-gene *tolC*-mediated pleiotropy. The mutations that result in mucoidy often give rise to other pleiotropic traits, including bacterial swimming motility [22,24]. Motility is an important dispersal mechanism for many bacteria, allowing cells to move from their original colony towards nutrients and symbiotic partners [25], and it is a trait associated with bacterial virulence [26].

Previous evolution experiments suggest that mutations that impact the mucoid phenotype may pleiotropically change motility, but the directionality of the effect varies by species and specific mutations [22,24]. For example, in *Pseudomonas aeruginosa* – which has a wild-type mucoid phenotype – non-mucoid mutants arose through *algT* (coding alternative sigma factor E) regulatory mutations that also increased flagellar motility [24]. Likewise, in experimentally evolved *Pseudomonas fluorescens* strain SBW25, unidentified mutations resulted in gain of mucoidy and loss of motility [22], again indicating antagonistic pleiotropy between the two traits. However, in another strain of *P. fluorescens*, MFE01, loss of mucoidy through mutations in *hcp1* (encoding a putative protein secretion apparatus) resulted in loss of motility, indicating positive pleiotropy [23]. Therefore, while mucoidy and its complexity may pose challenges to phage therapy, it may also co-occur with a cost to other fitness components.

Because of its relative complexity, we propose that mucoidy could be useful for understanding how pleiotropy is impacted by sets of genes and the genetics of evolutionary tradeoffs involving multiple loci. In our previous research on phage U136B resistance in which we characterized non-mucoid resistance [9], we noticed the occasional rise of bacteria with the distinctive mucoid morphology. However, in that work the mucoid phenotype did not appear frequently enough to be detected in our randomly sampled collection of bacterial mutants, which contained mutations impacting TolC and LPS [9]. To better understand the pleiotropic effects of mucoidy in *E. coli*, in this study we specifically selected and screened for mucoidy-based resistance to phage U136B in a high-replication experiment. We quantified two pleiotropic traits, phage resistance and motility, that resulted in an evolutionary tradeoff. This tradeoff was mediated by highly parallel changes to a small set of genes in the Rcs phosphorelay system, a multi-gene regulatory pathway that responds to cell-surface stress.

## Methods

### Overview of approach

To understand how mucoidy impacts phage resistance, we isolated and characterized independent phage-selected mucoid mutants of *E. coli* strain BW25113 using bacteriophage U136B. To quantify phage resistance of the potential mutants, we used phage-bacteria cross-streaks and efficiency of plaquing (EOP) assays. We then quantified bacterial swimming motility using 3 day motility time course assays in motility agar. To identify the genetic basis of the mutant phenotypes, we used whole-genome sequencing of all isolates, comparing their sequences to the wild-type bacterial reference genome.

### Bacterial and phage strains

BW25113 is a laboratory strain of *E. coli* K-12 that we routinely use to study interactions with phage U136B [9,27] and was obtained from the Coli Genetic Stock Center at Yale University (CGSC #7636). BW25113 *tolC* mutant RGB-040 (from our own collection) is used as a non-mucoid phage-resistant control [9]. Phage U136B is a 49 kb dsDNA phage with a curly tailed siphovirus morphology that relies on LPS and outer membrane protein TolC to infect BW25113 [9,27, 28].

### Bacterial and phage culture conditions

Bacterial and phage stocks were grown in shaken cultures in LB medium at 37 °C. Phages were generated by overnight growth on bacterial strain BW25113 followed by centrifugation and filtration of the supernatant through a 0.2 µm filter. Supernatant was stored at 4 °C for routine use. For long-term storage, all phage and bacterial stocks were stored at −80 °C in 20% glycerol. For phage plating, top (soft) agar overlays were made with 7.5 g agar l^–1^ in LB. The LB Miller [29] formulation was used for all growth conditions.

### Selection of phage-resistant isolates

We used traditional agar plate-based selection experiments to obtain phage-resistant colonies [30]. Each phage selection replicate was initiated from its own isolated colony of *E. coli* strain BW25113. Colonies were grown in LB broth overnight with shaking at 180 r.p.m. at 37 °C. Bacteria and phage were mixed at an m.o.i. of 10 (4×10^8^ p.f.u. of phage with 4×10^7^ c.f.u. bacteria) and spread onto the surface of an LB agar plate. Replicates were conducted in batches until a target collection of approximately 30 independent mucoid mutants was obtained. The spread plates were incubated at 37 °C for 2 days and then mucoid colonies – characterized by large size and enveloped in a viscous mass – and non-mucoid colonies were counted. From each plate, one mucoid colony was chosen at random, restreaked twice for isolation, grown overnight in LB and frozen at −80 °C in 20% sterile glycerol. Subsequent streak plates from the frozen samples sometimes revealed that the mucoid phenotype was not stable. We reasoned that because mucoid cells could be very sticky, they might be difficult to isolate from wild-type cells or non-mucoid phage-resistant cells, and so we conducted an additional five rounds of re-streaking for isolation, grew new overnight cultures and again froze samples at −80 °C in 20% glycerol. Subsequent growth of mucoid isolates was done from individual colonies in 4 ml LB broth overnight with shaking at 180 r.p.m. at 37 °C.

### Cross-streaks

First, 10 µl (1×10^7^ p.f.u. ml^–1^) of phage U136B was streaked in a vertical line down an LB agar plate. Bacterial samples were horizontally streaked across the phage line from either 1 µl of overnight culture or one isolated colony. The streaks were dried and incubated at 37 °C overnight. Sensitivity was scored when there was a lack of bacterial growth past the phage line. Resistance was scored when bacterial growth continued past the phage line.

### Efficiency of plaquing

Phage U136B high-titre stock (1×10^9^ p.f.u. ml^–1^) was serially diluted in a 1 : 10 series to 10^−8^ in LB broth, and 2 µl of each dilution was pipetted onto top agar overlays containing wild-type or mutant cells. To increase the assay limit of detection, we also spotted 10 µl of the undiluted phage onto one side of the plate. The spots were allowed to dry and incubated at 37 °C overnight. Values of p.f.u. ml^–1^ were calculated based on the highest dilution with plaquing. We calculated the EOP by dividing the p.f.u. ml^–1^ of phage U136B on each mutant by the p.f.u. ml^–1^ on wild-type bacteria.

### Motility assays

Motility assays were conducted in LB swim agar plates containing 0.3% agar. All plates were prepared with 20 ml volumes of LB agar for consistency across replicates. For each mutant or control, cultures were grown from individual colonies in LB broth at 37 °C overnight. Five microlitres of each overnight culture was pipetted onto the centre of a swim agar plate. The spots were allowed to dry and plates were incubated at 37 °C. The widest and narrowest diameters of each growth zone were measured after overnight incubation at 1, 2 and 3 days. We report the overall swim diameter as the mean of the widest and narrowest diameters for each mutant after 3 days of growth.

### Motility microscopy

Motility rainbow traces (‘ghost traces’) were made as done previously [31]. We sampled the outer front of bacteria from a motility agar plate with a 10 µl inoculating loop after 3 days of incubation. The sample was resuspended in 45 µl of Tris/Mg (TM) buffer, introduced into glass tunnel slides and viewed on an Olympus BH2 phase contrast microscope. Videos were recorded using Metamorph (v.7.1), trimmed to 5 s (75 frames), and their brightness and contrast were adjusted manually. ImageJ.JS (v.0.5.7) was used to generate rainbow traces using the macro ColorFootprint [32].

### Growth curves

Bacterial growth curve data were collected in LB in a 96-well plate format on a BioTek Synergy HGT plate reader with Gen5 software. Individual wells were filled with 180 µl LB and inoculated with 20 µl of overnight cultures. Samples were incubated at 37 °C. Every 3 min, samples were orbitally shaken for 5 s and OD_600_ was read after shaking.

### Genome sequencing

To prepare cultures for sequencing, 20 µl of −80 °C cryo samples were thawed and inoculated into 2 ml LB medium and incubated at 37 °C with shaking at 180 r.p.m. Whole-genome DNA was extracted using the Wizard Genomic DNA Purification Kit (Promega Cat. #A1120) according to the manufacturer’s instructions for Gram-negative bacteria, except that all steps were conducted at two-thirds the suggested volumes. DNA was sequenced at the Great Lakes Genomics Center (University of Wisconsin Milwaukee). Libraries were prepared with the Illumina DNA PREP kit with tagmentation (Cat. #20018705) using IDT UD Set A 10 bp indexes (Cat. #20027213) sequencing. DNA was sequenced on an Illumina NovaSeq 6000 producing 2×150 paired-end reads. Quality was assessed using MultiQC v1.0 dev0 [33]. Sequences were analysed with *breseq* v0.37.1 [34] using default parameters to identify changes compared to the wild-type reference genome (BW25113, Table 1, GenBank genome accession no. CP009273.1). The *breseq* output was manually curated to verify that all identified SNPs and small INDELS (<100 bp) appeared in 100% of reads. New junctions (e.g. those caused by movement of insertion sequences) were also manually verified and reported if they occurred in 100% of reads. Average genome coverage was 120.7× (range 69.4–247.1×).

**Table 1.** Results of whole-genome sequencing reveal the genetic basis for the mucoid phenotype

Mutant	Genome position	DNA mutation*	Amino acid change†(WT|codon|mutant)	Gene‡	Gene annotation in WT genome accession no. CP009273.1§
CS018	3 520 837	ΔGGC	ΔW337	*igaA* →	Negative regulator of the Rcs phosphorelay
	3 799 563	IS	Insertion in codon 289	*rfaG*	Glucosyltransferase I
CS019	2 312 296	C→T	E354K (GAA→AAA)	*rcsC* ←	Hybrid sensory kinase in two-component regulatory system with RcsB and RcsD
CS020	2 311 380	G→T	A659E (GCG→GAG)	*rcsC* ←	Hybrid sensory kinase in two-component regulatory system with RcsB and RcsD
CS021	3 520 915	C→T	P363L (CCG→CTG)	*igaA* →	Negative regulator of the Rcs phosphorelay
	4 348 384	T→G	E17D (GAA→GAC)	*cadA* ←	Lysine decarboxylase, acid-inducible
CS022	3 521 110	T→G	I428S (ATC→AGC)	*igaA* →	Negative regulator of the Rcs phosphorelay
CS023¶	972 225	G→A	L148L (CTG→CTA)	*mukB* →	Chromosome condensin
	1 191 733–1 206 916	Δ15184	Excision of e14 cryptic prophage	23 gene deletion	Δ(*ymfD, ymfE, lit, intE, xisE, ymfI, ymfJ, cohE, croE, ymfL, ymfM, oweE, aaaE, ymfR, beeE, jayE, ymfQ, stfP, tfaP, tfaE, stfE, pinE, mcrA*)
	2 966 200	C→T	G341E (GGG→GAG)	*lplT* ←	Lysophospholipid transporter
	3 519 808	IS	Intergenic (–300/–20), *igaA* promoter	*nude/igaA*	Adenosine nucleotide hydrolase/negative regulator of the Rcs phosphorelay
CS024	2 312 210	T→G	L382F (TTA→TTC)	*rcsC* ←	Hybrid sensory kinase in two-component regulatory system with RcsB and RcsD
CS025	2 112 139	T→A	Intergenic (−254/+22)	*wcaK* ←/← *wzxC*	Colanic acid biosynthesis protein/putative colanic acid exporter
	3 521 467	T→A	L547Q (CTG→CAG)	*igaA* →	Negative regulator of the Rcs phosphorelay
CS026	518 671	(T)_9→10_	Intergenic (+385/–46)	*ybbP* →/→ *rhsD*	Putative ABC transporter permease/Rhs family putative polymorphic toxin
	2 311 926	A→G	V477A (GTC→GCC)	*rcsC* ←	Hybrid sensory kinase in two-component regulatory system with RcsB and RcsD
CS027	3 521 110	T→G	I428S (ATC→AGC)	*igaA* →	Negative regulator of the Rcs phosphorelay
	3 909 274	A→G	L20P (CTG→CCG)	*atpC* ←	F1 sector of membrane-bound ATP synthase, epsilon subunit
CS028	3 520 695	T→G	Y290D (TAT→GAT)	*igaA* →	Negative regulator of the Rcs phosphorelay
CS029	394 594	C→T	V66M (GTG→ATG)	*yaiY* ←	DUF2755 family inner membrane protein
	961 542	C→T	P589P (CCC→CCT)	*ycaI* →	ComEC family inner membrane protein
	2 005 409	C→A	Pseudogene (113/678 nt)	*yedN* ←	Pseudogene, IpaH/YopM family
	2 311 938	A→C	F473C (TTC→TGC)	*rcsC* ←	Hybrid sensory kinase in two-component regulatory system with RcsB and RcsD
	3 799 566	IS	Insertion in codon 289	*rfaG*	Glucosyltransferase I
CS030	3 521 110	T→G	I428S (ATC→AGC)	*igaA* →	Negative regulator of the Rcs phosphorelay
	3 652 331	IS	Intergenic (+81/–258)	*gadE/mdtE*	gad regulon transcriptional activator/anaerobic multidrug efflux transporter, ArcA-regulated
CS031	2 311 908	G→A	T483M (ACG→ATG)	*rcsC* ←	Hybrid sensory kinase in two-component regulatory system with RcsB and RcsD
HT018	3 521 829	A→C	S668R (AGC→CGC)	*igaA* →	Negative regulator of the Rcs phosphorelay
HT019	2 308 195	C→A	A410E (GCG→GAG)	*rcsD* →	Phosphotransfer intermediate protein in two-component regulatory system with RcsBC
HT020	496 184	G→A	G202S (GGT→AGT)	*gsk* →	Inosine/guanosine kinase
	2 307 135	A→C	T57P (ACC→CCC)	*rcsD* →	Phosphotransfer intermediate protein in two-component regulatory system with RcsBC
	3 576 391	IS	Insertion in codon 390	*yhhZ*	Putative Hcp1 family polymorphic toxin protein; putative colicin-like DNase/tRNase activity
	3 944 978	C→T	Pseudogene (77/663 nt)	*ilvG* →	Pseudogene, acetolactate synthase 2 large subunit, valine-insensitive; acetolactate synthase II, large subunit, cryptic, interrupted
HT021	3 520 231	T→C	L135P (CTG→CCG)	*igaA* →	Negative regulator of the Rcs phosphorelay
HT022	3 519 850	(T)_7→6_	Stop codon at position 24	*igaA*→	Negative regulator of the Rcs phosphorelay
HT023	2 310 642	T→A	N905I (AAT→ATT)	*rcsC* ←	Hybrid sensory kinase in two-component regulatory system with RcsB and RcsD
HT024	2 307 606	A→C	T214P (ACA→CCA)	*rcsD* →	Phosphotransfer intermediate protein in two-component regulatory system with RcsBC
	3 800 126	IS	Insertion in codon 101	*rfaG*	Glucosyltransferase I
HT025	3 521 755	T→C	L643P (CTT→CCT)	*igaA* →	Negative regulator of the Rcs phosphorelay
HT026	3 521 110	T→G	I428S (ATC→AGC)	*igaA* →	Negative regulator of the Rcs phosphorelay
HT027	2 311 938	A→G	F473S (TTC→TCC)	*rcsC* ←	Hybrid sensory kinase in two-component regulatory system with RcsB and RcsD
HT028	3 520 071	Δ186 bp	Δ62 aa at position 82	*igaA* →	Negative regulator of the Rcs phosphorelay
HT029	2 499 658	G→A	R152C (CGC→TGC)	*ypdF* ←	Xaa-Pro aminopeptidase
	3 104 868	G→T	Pseudogene (582/963 nt)	*yghE* ←	Pseudogene, secretion pathway protein, L-type protein homology; putative general secretion pathway for protein export (GSP)
	3 520 162	T→C	L112P (CTC→CCC)	*igaA* →	Negative regulator of the Rcs phosphorelay
	4 130 618	A→C	I511S (ATC→AGC)	*frwA* ←	Putative PTS enzyme, Hpr component/enzyme I component/enzyme IIA component
	4 322 433	Δ1 bp	Frame shift at position 219	*basS* ←	Sensory histidine kinase in two-component regulatory system with BasR
HT030	3 520 609	G→C	R261P (CGC→CCC)	*igaA* →	Negative regulator of the Rcs phosphorelay
HT031	1 123 654	A→T	T120T (ACA→ACT)	*murJ* →	Putative peptidoglycan lipid II flippase
	3 519 808	IS	Intergenic (–300/–20), *igaA* promoter	*nudE/igaA*	Adenosine nucleotide hydrolase/negative regulator of the Rcs phosphorelay

1*Indicates wild -type → mutant nucleotide. In the case of an insertion sequence disruption, ‘IS’ is indicated.

2†Letters indicate the standard amino acid code and numbers the amino acid position of the protein. In the case of pseudogenes, place in the DNA sequence is shown. In the case of intergenic regions, position relative to flanking gene(s) is shown. For mutant CS023, the e14 prophage deletion is noted.

3A‡Arrows in the Gene column indicate orientation of the gene in the genome. *igaA* is also called *yrfF,* the gene name used in the reference genome.

4§*igaA* is annotated in CP009273.1 as an ‘inner membrane protein’ but updated here to reflect newer research. The *rcsC* annotation indicates interaction with YojN, updated here to RcsD.

5¶Four additional putative SNPs at the e14 attachment site were called by *breseq* but are not *de novo* mutations; these are instead likely probably due to DNA rearrangement during integarase-mediated e14 cryptic prophage excision [50]: 1 191 676 C→T, 1 191 688 C→T, 1 191 701 T→C, and 1 191 703 A→G.

### Statistical analysis

All statistical analysis was done with R [35] using RStudio [36]. To test whether the mutants had unique phage resistance phenotypes (measured by EOP), we used general linear models accounting for batch effects. To test whether the mutants had unique swimming motility phenotypes (quantitative diameters on swim agar plates), we used general linear models accounting for batch effects. We performed post-hoc *t* tests to test if individual mutant phenotypes were different from that of the wild-type reference. For EOP, we used one-sample *t* tests and compared to the fixed wild-type reference EOP of 0. For motility, we used two-tailed *t* tests and compared to the measured swimming diameters of the wild-type reference. For all *t* tests, we corrected for multiple comparisons using the Holm–Bonferroni method, in which *P*-values are ranked in ascending order and alpha for each test is adjusted by the formula 0.05/(no. of comparisons – Rank+1). To test whether the growth rate of a mutant predicts its mean motility, we used a linear model (LM) [which assumes a Gaussian (normal) distribution and uses the identity link function] of growth rate as a function of mean motility with all 27 mucoid mutants plus the wild-type reference strain. To test whether the EOP of a mutant predicts its mean motility, we used linear models of log(EOP) as a function of mean motility and analysed the dataset both with increasing levels of conservative exclusion: (1) with the ancestral wild type included, (2) without the ancestral wild-type included, (3) without *rfaG* mutants, and (4) without ancestral and without *rfaG* mutants. All data figures were generated in R using RStudio [36] and ggplot2 [37]. Growth curves were analysed using the R package gcplyr [38].

### Data availability

Genomic sequence data are available on the publicly available Sequence Read Archive (SRA) BioProject no. PRJNA608759. All accession numbers are listed in Data File S2, available in the online version of this article.

## Results

### Mucoid mutants occur at lower frequencies than non-mucoid mutants

**Fig. 1.**
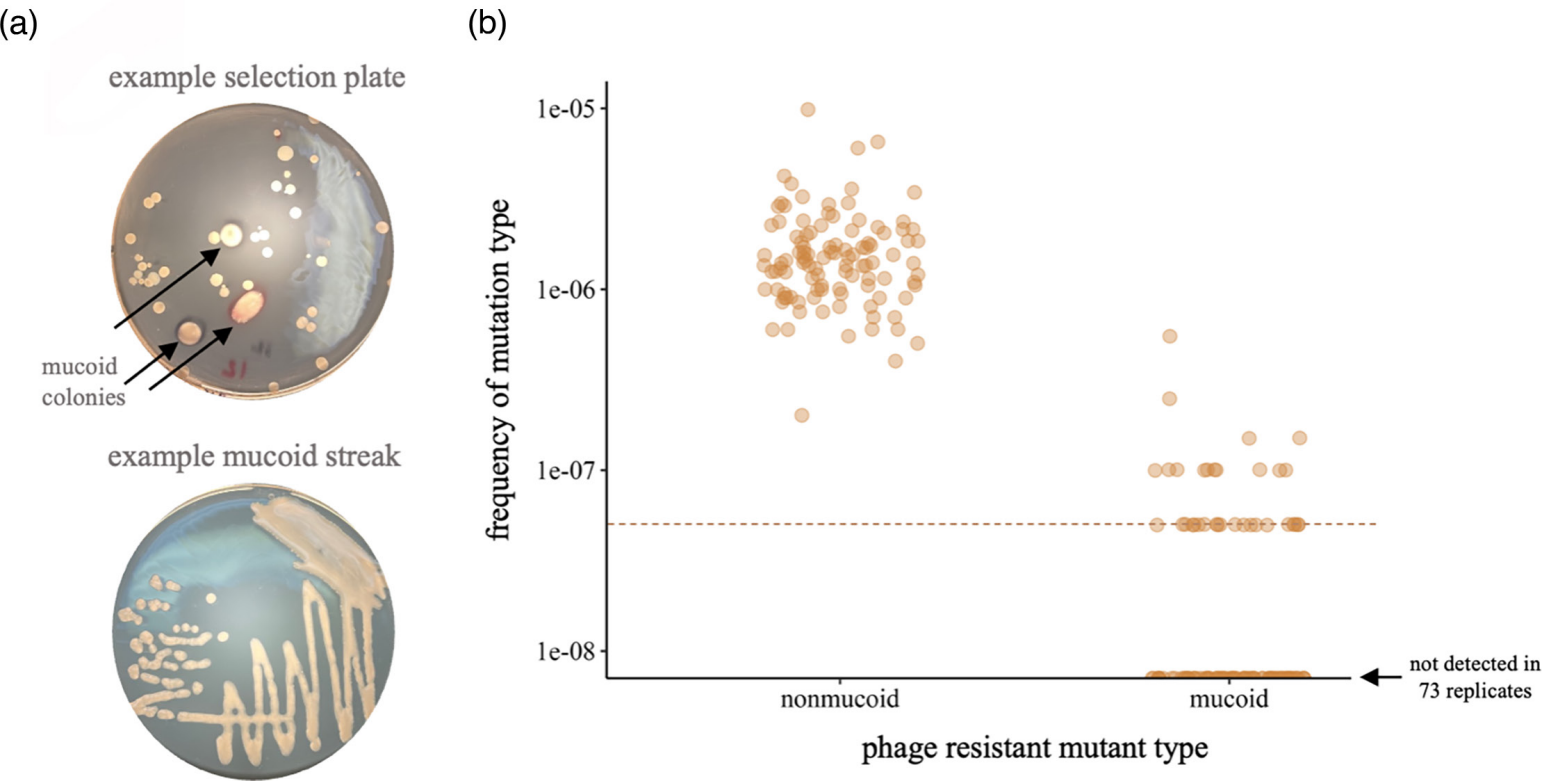
Mucoid mutants appear under phage U136B selection. (a) Example selection and isolation of phage-resistant mucoid mutants, which appear large and enveloped in a shiny, viscous mass. (b) Frequencies of phage-resistant mutants with mucoid and non-mucoid colony morphologies. Individual data points represent the frequencies of each mutant colony morphology in the 107 replicates of the selection experiment. The dashed line indicates the limit of detection at 5×10^−8^. Data points have a horizontal jitter added for visibility.

To characterize mucoid genotypes that arise under selection by phage U136B, we conducted a selection experiment that revealed mucoid and non-mucoid mutants. After selection, mucoid colony morphology was the least common mutant type, appearing in a minority of replicates (34/107) (Fig. 1). All replicates contained non-mucoid colonies, as we previously reported to include *tolC* and LPS-related mutations [9].

### Isolation of mucoid mutations

We next randomly sampled a mucoid colony from each of the 34 mucoid-containing replicates. From the 34 original replicates containing mucoid colonies, this process resulted in 28 candidate mucoid mutants that had stable mucoid phenotypes (Table 1).

### Cross-streaks confirm phage resistance

To confirm that the isolates were resistant to phage U136B, we used cross-streak tests (Fig. S1). One isolate (HT030) was consistently sensitive to phage U136B and began displaying a non-mucoid phenotype, so it was omitted from further analysis. The other 27 mutants were consistently resistant across many replicates (*N*=2 biological replicates from overnight cultures and *N*=10 biological replicates from individual colonies). Only one isolate (HT029) had one replicate that appeared sensitive, but upon repeating all other tests it showed resistance.

### Efficiency of plaquing reveals variation in phage resistance levels

**Fig. 2.**
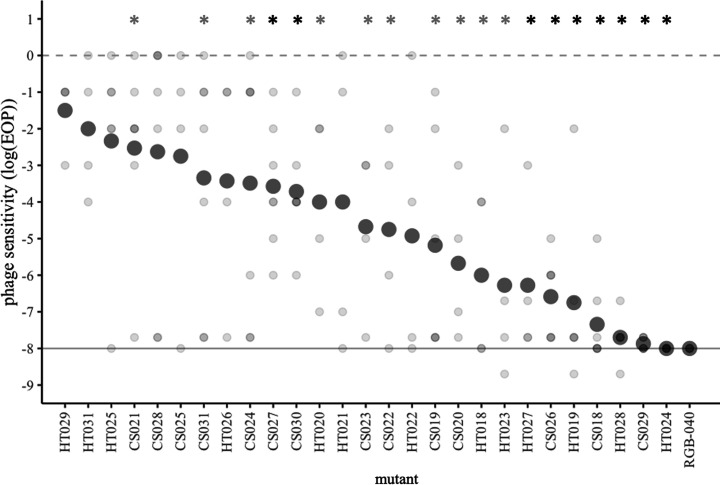
Efficiency of plaquing assays reveal variation in quantitative phage resistance. Light grey points represent individual replicates (*N*=4–7 per mutant). Dark grey points represent the mean of log(EOP) for all replicates. The solid line at 10^−8^ indicates the typical limit of detection of the assay; for any point where EOP could not be detected, it is conservatively plotted and analysed at this limit. The dashed line indicates the log(EOP) of the wild-type strain, which by definition is 0. Asterisks indicate results of one-sample *t* tests compared to the wild-type reference, alpha=0.05: a grey asterisk (*) indicates *P*<0.05 before correction for multiple comparisons; a black asterisk (*) indicates significance after Holm–Bonferroni correction for multiple comparisons. RGB-040 is a non-mucoid *tolC* mutant control.

Cross-streaks provide a qualitative indicator of phage resistance, but they do not provide the quantification needed to characterize different levels of phage resistance. We used EOP assays to quantitatively characterize phage resistance and to obtain plaque phenotypes, which can reveal additional information about a given host’s susceptibility to a phage. We found that all 27 of the mucoid mutants had reduced mean EOP, indicating overall increased phage resistance (Fig. 2). Several mutants (HT028, HT024 and CS029) consistently had extremely low or undetectable plaque formation, indicating complete phage resistance, while all other mutants showed varying degrees of partial resistance relative to the wild-type. A control *tolC* mutant (RGB-040) consistently showed no plaquing. Across mucoid mutants, the mean EOP (bold points in Fig. 2) varied by several orders of magnitude, suggesting that our 27 mutants represent unique phage-resistance phenotypes. To test this, we used a general linear model to account for batch effects (*F*_4, 116_=6.9, *P*<0.00001) and found a significant effect of mutant on EOP (*F*_26, 116_=4.4, *P*<0.00001). For any given mutant, the EOP could vary many orders of magnitude across biological replicates (lightly coloured points in Fig. 2). This replicate-to-replicate variation suggested that the mucoid morphology can be phenotypically variable, where a specific genotype can give rise to some cultures that are highly phage resistant whereas other replicate cultures of the same genotype appear to be more phage sensitive. For all replicates of mucoid mutants where plaques did appear they were faint or hazy, compared to the clear plaques observed on the wild-type, BW25113 (Fig. S2).

### Mucoid mutants have diminished swimming motility

**Fig. 3.**
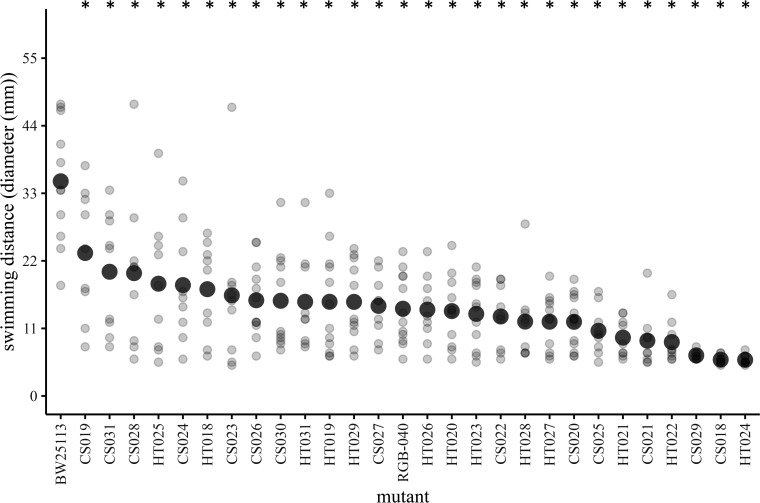
Mucoid mutants have diminished swimming motility. Light grey points represent individual replicates (*N*=9–12 per mutant). Dark grey points represent the mean of all replicates. Asterisks indicate results of *t* tests compared to the wild-type reference (*P*<0.05 with Holm–Bonferroni correction for multiple comparisons). RGB-040 is a non-mucoid *tolC* mutant control.

Since mucoidy could interfere with cell-surface structures involved in motility, we next quantified the 27 mucoid mutants’ swimming speed. All mucoid mutants had reduced mean swim diameter compared to the wild-type, indicating that mucoid-based phage resistance comes at a general cost to swimming motility (Fig. 3). Swimming ability varied among the mutants, and as with the EOP assays, many mutants varied from replicate-to-replicate (general linear model: *F*_28, 260_=9.1, *P*<0.00001 for mutant effect, *F*_3, 260_=28.6, *P*<0.00001 for day effect). We qualitatively verified these results by analysing the variation in swimming ability of individual cells using phase contrast microscopy of samples taken from the outer edge of the motility assay spots (Fig. S3). We also tested whether the reduction in swimming ability could be due to a reduction in growth rate. We found that many of the mucoid mutants (18/27) have a faster growth rate than the ancestor, opposite to the direction we would expect if mucoidy imposed a growth rate defect (Fig. S4A, B). We observed no effect of growth rate on swimming distance (LM, *P*=0.74, *R*^2^=0.004) (Fig. S4C).

### Whole-genome analysis

**Fig. 4.**
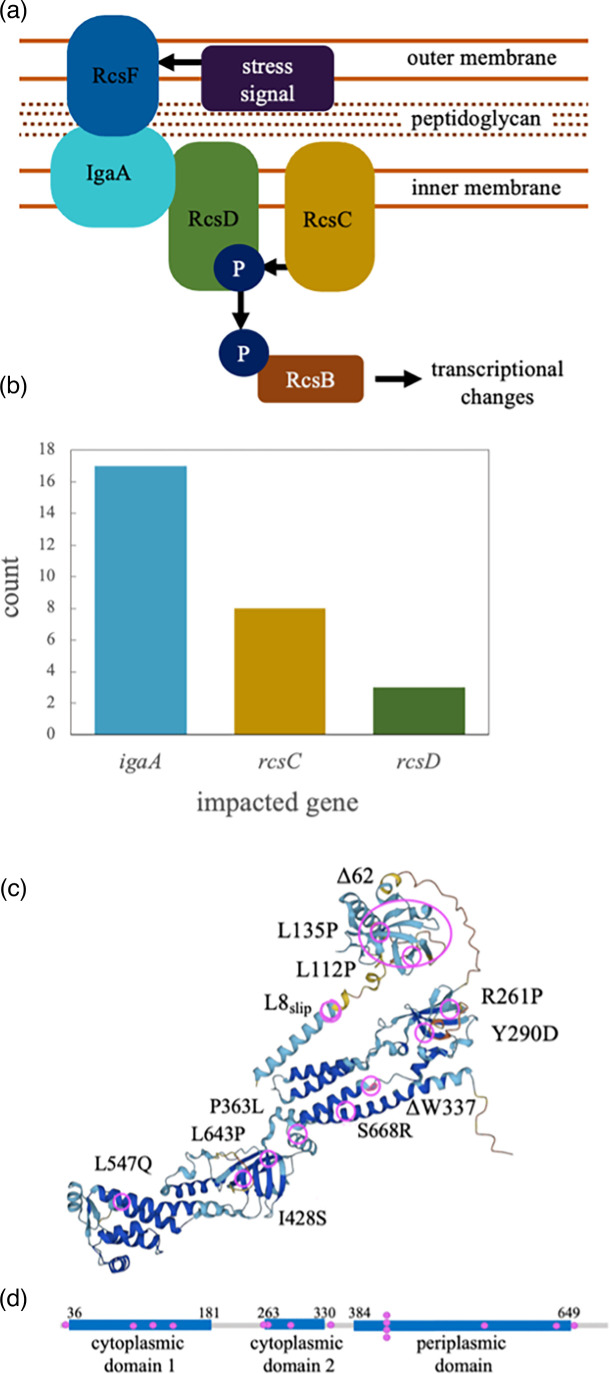
Parallel mutations of the Rcs phosphorelay system. (a) The Rcs phosphorelay system conveys signals of stress through the outer and inner membranes via IgaA, RcsD and RcsC. IgaA conveys the stress signal from RcsF to RcsD, resulting in phosphorylation of the response regulator RcsB. Schematic in (a) is based on work by Wall *et al*. [39]. (b) Phage selection results in parallel changes to genes encoding IgaA, RcsC and RcsD. (c) Position of mutations affecting the IgaA protein. Protein structure was predicted using Alphafold [51] and amino acid positions were manually annotated. (d) Position of mutations in the linear IgaA amino acid sequence.

To identify the genes involved in the resistance and motility phenotypes, we sequenced the genomes of each of the 27 mucoid mutants. We also included HT030, which had been omitted earlier based on its unstable phenotypes (*N*=28 total). The majority of the mutants (16/28) contained only a single mutation, such that we can directly link the mutant genotype to the corresponding phenotype of that isolate (Table 1). We observed highly parallel changes to genes involved in membrane signalling and transport. In particular, genes or regulatory regions of the Rcs phosphorelay were mutated in 28/28 sequenced mutants (27/27 phenotypically mucoid mutants). This pathway conveys signals of envelope and peptidoglycan stress to the inner membrane and then to a cytoplasmic transcriptional regulator [39] (Fig. 4a).

We observed mutations in the three Rcs phosphorelay genes encoding inner membrane proteins: *igaA* (also known as *yrfF*, 15 mutants with coding-sequence mutations and two with promoter mutations), *rcsC* (eight mutants) and *rcsD* (three mutants) (Fig. 4b, Table 1). Of the 15 *igaA* ORF mutations, 14 have predicted substitutions or non-frameshifting deletions (Fig. 4c). One *igaA* mutation results in a predicted early stop codon due to a polyT_7_ to polyT_6_ mutation. Isolate HT030 (omitted from our earlier assays based on inconsistent mucoidy and phage sensitivity) contained an *igaA* mutation.

No isolates contained mutations in genes previously determined to be essential for phage U136B infectivity [9], including *tolC* and LPS synthesis genes *rfaC*, *rfaD*, *rfaE* and *rfaP*. Some mutants did contain additional mutations, including three mutants with mutations in a different LPS synthesis gene, *rfaG*, which is not essential for U136B infection [9]. Mutations also occurred in other genes not known to be related to phage susceptibility (Table 1).

### Relationship between phage resistance and swimming motility

**Fig. 5.**
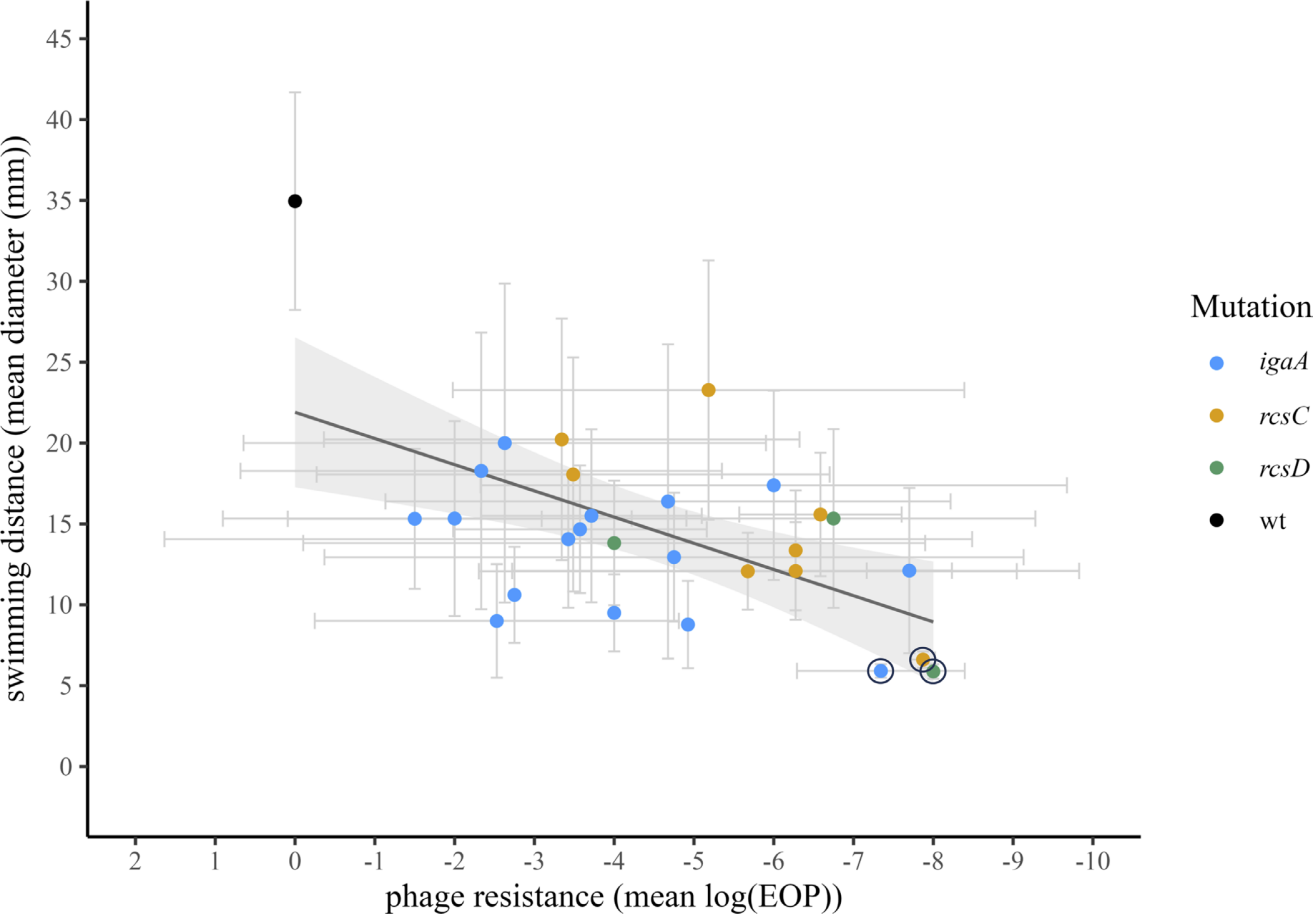
Relationship between phage resistance and motility. Grey error bars indicate 95% confidence intervals for each point. The log(EOP) of the wild type (black point) is by definition 0, so it does not have horizontal error bars. The grey shaded band indicates 95% confidence intervals around the linear model fit (solid black line) including the wild-type and all mutants (*P*<0.01, *R*^2^=0.31, but see main text and Table S1 for further discussion and other models). The three mutants containing *rfaG* mutations are indicated with black rings.

Rcs phosphorelay mutations that confer mucoid phage resistance resulted in reduced motility (Fig. 3), but does the *degree* of resistance correlate with the *degree* of motility defect among mucoid mutants? We found that the mean EOP of a mutant significantly predicts its mean motility (LM *F*_1,26_=12.9, *P*=0.001), including when the ancestral wild type is not included (LM *F*_1, 25_=5.7, *P*=0.025) or when the *rfaG* mutations (which may be additionally associated with phage resistance) were excluded (LM *F*_1,23_=4.9, *P*=0.037) (Fig. 5 and Table S1). However, the mean EOP of a mutant did not significantly predict its mean motility when both wild type and the *rfaG* mutants were omitted (LM *F*_1,22_=0.33, *P*=0.575) (Table S1).

## Discussion

### A case of pleiotropy with a continuum of effects

We isolated a set of mutants that demonstrated a tradeoff between phage resistance and bacterial motility. These mutants contained a variety of genetic mutations, but all contained mutations impacting three genes of the Rcs phosphorelay that regulates expression of mucoidy and other phenotypes. Therefore, these alleles have far-reaching effects, including all genes regulated by the pathway. Because distinct mutations may cause different slight changes to the Rcs proteins, they may vary in their effects on the efficiency of phospho-transfer or signal transduction, resulting in a continuous distribution of changes to transcriptional regulation. This in turn could result in a continuum of phenotypic effects for both mucoidy and motility. Indeed, we observed a range of phenotypes for both phage resistance and motility (Figs2, 3  5).

In contrast, previous work in the study of antagonistic pleiotropy has often identified loci that display what we term ‘all-or-nothing’ pleiotropy, which we define as when a change in one trait results in a change to another trait, and the magnitudes of the changes are extreme. We predict that all-or-nothing pleiotropy may be common when caused by large changes to single structural genes. Large changes – such as frameshift mutations or indels – could potentially knock out gene function. If a gene has more than one function, then its protein-level pleiotropic effects would severely affect those functions, resulting in a complete loss or gain of a phenotype. These types of changes have long been reported in the microbiology literature, although usually not termed pleiotropy. In some cases, the traits were quantitively measured, but the phenotypes are so extreme that there is little to no distribution of the values. For example, Bradbeer *et al*. studied *btuB* mutants that have undetectable levels of phage BF23 adsorption, and levels of BtuB (the vitamin B12 transporter) fall below the detection limit [40]. In other instances, one of the pleiotropic traits may have a quantitative distribution but the other trait does not have variation. For example, *tolC* mutants have varying sensitivities to bacteriocin colicin E1, but they have an extremely high, narrow range of phage TLS resistance near or at the detection limit (EOPs of 10^−7^ to 10^−9^) [41].

We predict that continuous pleiotropy may have a distinct genetic basis from all-or-nothing pleiotropy. For instance, continuous pleiotropy may be more common when caused by mutations of small effect, especially in genes involved in regulatory pathways that impact the expression of many genes and their resulting traits. For example, in this case the changes that result in increased mucoidy also impact reduced motility. The reduction in motility probably occurs through reduced expression of the flagellar master regulator operon *flhDC*, which is negatively regulated by the Rcs phosphorelay [42,43]. Alternatively, although not the case in our work, continuous pleiotropy could be caused by mutations in genes underlying polygenic traits, such that changes in one or more genes could result in a quantitative distribution of phenotypes. Continuous pleiotropy may also be more common when the strength of selection is lower, thereby allowing partially functional phenotypes to be competitive. Continuous pleiotropy could also be either antagonistic (resulting in a trade-off, as in this study) or beneficial (resulting in a trade-up). These and other predictions will be productive avenues for future research in pleiotropy.

Our results also suggest that while pleiotropy may result in a continuum of phenotypic effects for two traits, the degree of those effects is not necessarily correlated. In our data, we do see a continuum of phenotypes for both phage resistance and bacterial motility (Figs2, 3  5). Therefore, these mutants do not represent a case of all-or-nothing pleiotropy. Instead, changes to the Rcs phosphorelay result in continuous changes to both phage resistance and motility, even though the two traits are not necessarily correlated to one another when the wild-type and specific mutants are excluded from analysis (Table S1).

### Implications for applied phage biology

Beyond this basic science, U136B also serves as a model system for understanding phage steering and has potential as a phage therapeutic. To develop phage-based treatments for bacterial pathogens, it is essential to understand both the intended and unintended evolutionary consequences of phage selection. Here we found that mucoidy arises readily but less often than other U136B resistance mutations (including *tolC* and LPS-related mutations, Fig. 1 [9]). Although mucoidy is an undesirable outcome of therapy that could enable increased bacterial virulence, reduction to bacterial motility could be a favourable outcome of phage therapy. In previous work, we found that during experimental evolution (with serial passaging), only *tolC* mutations reached high frequencies, and we did not observe the evolution of mucoid mutants during serial passaging [9]. Future work would be needed to assess how commonly mucoidy evolves *in vivo* compared to *tolC* and LPS-related mutations.

### Mucoid stability

We frequently noted phenotypic instability among our mucoid mutants. For example, mucoid colonies may take 2 days to display the mucoid phenotype, but this phenotype would sometimes disappear during standard storage of agar plates at refrigeration temperatures. Early on in our experimentation, this variability complicated the isolation of pure mucoid colonies, and so we conservatively omitted some isolates from further analysis if they did not reliably produce mucoid colonies upon restreaking (a traditional metric of a microbial trait not being heritable). Indeed, even after two rounds of purification, we continued to see some variation, and so we streaked for isolation an additional five times (see Methods).

One peculiar result from this work was that – even after the additional rounds of isolation – the HT030 (Table 1) mutant later stopped displaying the mucoid and phage-resistant phenotypes, and so we omitted this strain in our phenotyping experiments. However, later in the project we were curious about a possible reversion mechanism (as suggested previously [22]) and so we included HT030 in the subsequent genotyping experiment, where we discovered a mutation in *igaA* (R261P, arginine to proline at amino acid position 261, Table 1). It is possible that this mutation results in an unstable phenotype that frequently appears non-mucoid and phage sensitive. Alternatively, a gene duplication event may have relieved the defect in the pathway, resulting in loss of the mucoid phenotype even though the original mutation remains in the genome. However, the coverage of our HT030 sequences at the *igaA* locus was not twice that of the rest of the genome (215× compared to the genome average of 170×), indicating that gene duplication was unlikely.

Past work on mucoid mutants suggested high reversion rates from mucoid to non-mucoid [22]. However, those results may have been confounded by use of incompletely purified bacterial isolates (indicated by the presence of phage in some samples), such that non-mucoid reversion may have actually been due to the re-appearance of wild-type bacteria from mixed colonies. Because we extensively purified our isolates, it is unlikely that our isolates were mixed genotypes.

We predict that the *phenotypic* instability of the mucoid phenotype may also result in instability of the associated traits of phage resistance and motility. Indeed, we observed high degrees of variance in those traits (Figs2  3). To minimize variation, we took care to conduct assays under identical conditions (always measuring 20 ml of agar into Petri plates, using the same incubators and incubation times, etc.). This consistency in our methodology suggests that subtle nuances of the laboratory environment might result in the observed phenotypic variability. Future work to identify those nuances might help to characterize the nature of genetic regulation of mucoidy – if the nuances indeed can be identified at all. However, it is also possible that the mutant phosphorelays are highly sensitive to environmental stress sensing, resulting in the variance we have observed. In either case, one way to account for such variation is with well-replicated quantitative experiments and statistical analysis that shows strong effect sizes with statistical significance, as we have done here.

### Unique mutations of the Rcs phosphorelay genes

All 28 of the mutants had mutations in genes impacting the Rcs phosphorelay pathway (*igaA*, *rcsC* and *rcsD*, Fig. 4, Table 1). These mutations are useful for better understanding the role of IgaA as a negative regulator of the Rcs phosphorelay, a key stress-sensing pathway important to intrinsic beta-lactam antibiotic resistance [44]. Our results are consistent with work showing that IgaA and RcsD interact through (1) their periplasmic domains, and (2) IgaA’s cytoplasmic domain and RcsD’s PAS-like domain [39]. We observed *igaA* mutations in both the periplasmic domain (amino acid positions 384–649) and the cytoplasmic domains (amino acid positions 36–181, 263–330). Two of the *rcsD* mutations (T57P, T214P) were in the periplasmic domain, and one mutation (A410E) was directly within the PAS-like domain involved in phosphotransfer to RcsC (Fig. 4d, Table 1). In some cases, the IgaA mutations occurred just outside the regions of the protein previously known to interact with RcsD (Fig. 4d, the cytoplasmic and periplasmic domains). This result suggests that subtle changes to the secondary structure of IgaA may impact stability or interaction of other parts of the protein that are required for its regulatory function.

In most of our isolates, there was only one mutation, so we can directly link the genetic change to the changed mucoid and phage resistance phenotypes (Table 1). In some cases, mutants had more than one mutation. Although multiple mutations are somewhat unexpected in fluctuation assays, we hypothesize that additional mutations rode along with the mucoid phage resistance mutations for three reasons. First, we streaked for isolation an additional five times in order to ensure purity, resulting in additional time for secondary mutations to arise. Second, we hypothesize that the mucoid mutants – which have cells coated in an adhesive layer of polysaccharide – may be more likely to have phages sticking to them during subsequent streaks for isolation. During the extended isolation, it is possible that some isolates faced continued selection pressure by any ‘stuck’ phages before they were purified. For instance, this may explain the occurrence of *rfaG* mutations in three of the mutants. We previously found that on its own, knocking out *rfaG* conferred a very small amount of phage resistance [9]. Here, we find that double mutants containing an Rcs phosphorelay mutation and an *rfaG* mutation have very high levels of resistance (mutants CS018, CS029 and HT024, Fig. 2, Table 1), which could suggest epistasis between these alleles. Finally, it is possible that during the extended isolation, some lineages picked up compensatory mutations that alleviated fitness costs of the mucoid-causing mutations.

### Essentiality of IgaA

Previous work has suggested that *igaA* is essential, yet one of our mutants contains a frameshift mutation that should severely truncate the IgaA protein. Our mutant HT022 has a single base pair deletion that mutates a poly(T)_7_ tract into a poly(T)_6_ tract. This deletion is predicted to result in a frameshift and early stop codon at amino acid position 24, equivalent to a near-total loss of the 711 aa protein. In contrast, previous work in *Salmonella enterica* Typhimurium strain SL1344 found that *igaA* was essential unless other compensatory mutations were already present in the other phosphorelay genes *rcsC*, *rcsD* or *rcsB* [45]. Likewise, *igaA* is required in *E. coli* strain MG1655 in a genotype-by-genotype-dependent manner, specifically when the other components of the Rcs system are fully functioning [39]. Even in BW25113, *igaA* was determined to be essential using transposon-directed insertion site sequencing, although in slightly different culture conditions than what we used (LB vs LB Miller) [46]. Why is the HT022 *igaA* mutation not lethal? One possibility is that RNA polymerase slippage along the poly(T)_6_ tract allows sufficient gene expression to maintain viability [47]. Alternatively, strain differences may result in different requirements for *igaA* (Discussion Note S1).

Interestingly, the *igaA* promoter mutations (in isolates CS023 and HT031) were both accompanied by non-Rcs mutations (Table 1). It is possible that those additional mutations may reflect compensatory adaptation for the fitness effects of reduced *igaA* expression. These mutants would be good candidates for future work to understand how IgaA interacts with the cell wall and membrane during Rcs phosphorelay regulation. However, as one of *E. coli*’s most complicated two-component regulatory systems [48,49], fully understanding the Rcs phosphorelay’s molecular control is well outside the scope of the present study.

### Conclusion

We have identified and characterized a complete set of novel mucoid mutations in the Rcs phosphorelay that confer partial phage resistance and reduced motility. The relationship between these traits follows a pattern of continuous, rather than all-or-nothing, antagonistic pleiotropy. This collection of mutants will be useful for future studies on the mechanisms of pleiotropy and the specific molecular interactions among phosphorelay genes.

## supplementary material

10.1099/mic.0.001491 Uncited Supplementary Material 1.

10.1099/mic.0.001491 Uncited Supplementary Material 2.

## Abbreviations

EOP: efficiency of plaquing
LM: linear model
LPS: lipopolysaccharide
SRA: Sequence Read Archive
