# Supplementary material for: A tradeoff between bacteriophage resistance and bacterial motility is mediated by the Rcs phosphorelay in Escherichia coli
Source: Microbiology (Reading). 2024 Aug 28;170(8):001491. doi: 10.1099/mic.0.001491 (PMC11541549; doi:10.1099/mic.0.001491)
Supplement: Uncited Supplementary Material 1. [file mic-170-01491-s001.pdf]

Supplement for:
A Tradeoff Between Bacteriophage Resistance and Bacterial Motility is Mediated by the Rcs Phosphorelay in *Escherichia coli*

Alita R. Burmeister, Harleen Tewatia, and Chloé Skinner

SUPPLEMENTAL DISCUSSION

Note 1. Essentiality of *igaA*: We observed one mutation that could potentially result in a frameshift mutation in igaA (poly(T)$_7$ to poly(T)$_6$ in mutant HT022). In the main text, we propose that RNA polymerase slippage allows sufficient *igaA* expression to maintain viability. Alternatively, strain-to-strain differences in Rcs phosphorelay genes may result in different requirements for *igaA*. To test this, we used pairwise blast (NCBI) to align the Rcs phosphorelay genes of *E. coli* BW25113 (used in this study, Genbank Accession #CP009273.1) to *E. coli* MG1655 (Genbank Accession #U00096.2). We found that all four genes were identical in the two strains (Table S2), suggesting that either *igaA* is essential in BW25113 just like MG1655, or that *igaA* is non-essential due to differences elsewhere in the genome.

SUPPLEMENTAL TABLES

Table S1. Results for relationship between phage resistance and motility using linear models with increasing levels of conservative exclusion.

**Model**	***F***	***p***	**R^2**
All mucoid mutants and wild type	$F_{1, 26} = 12.86$	0.001	0.331
Excluding wild type	$F_{1, 25} = 5.689$	0.025	0.185
Excluding mutants with *rfaG* mutations	$F_{1, 23} = 4.893$	0.037	0.175
Excluding wild type and mutants with *rfaG* mutations	$F_{1, 22} = 0.325$	0.575	0.015

Table S2. Comparison of Rcs phosophrelay genes between BW25113 and MG1655, where *igaA* has been shown to be essential. Results are from nucleotide BLAST comparison of the coding nucleotide sequence of each gene from each strain.

Gene	*E. coli* BW25113 Accession #CP009273.1 Genome location	*E. coli* MG1655 Accession #U00096.2 Genome location	% identity
rcsB	2,309,656 - 2,310,306	2,314,199 - 2,314,849	100%
rcsC	2,310,506 - 2,313,355	2,315,049 - 2,317,898	100%
rcsD (*yojN*)	2,306,967 - 2,309,639	2,311,510 - 2,314,182	100%
igaA	3,519,828 - 3,521,963	3,524,491 - 3,526,626	100%

SUPPLEMENTAL FIGURES

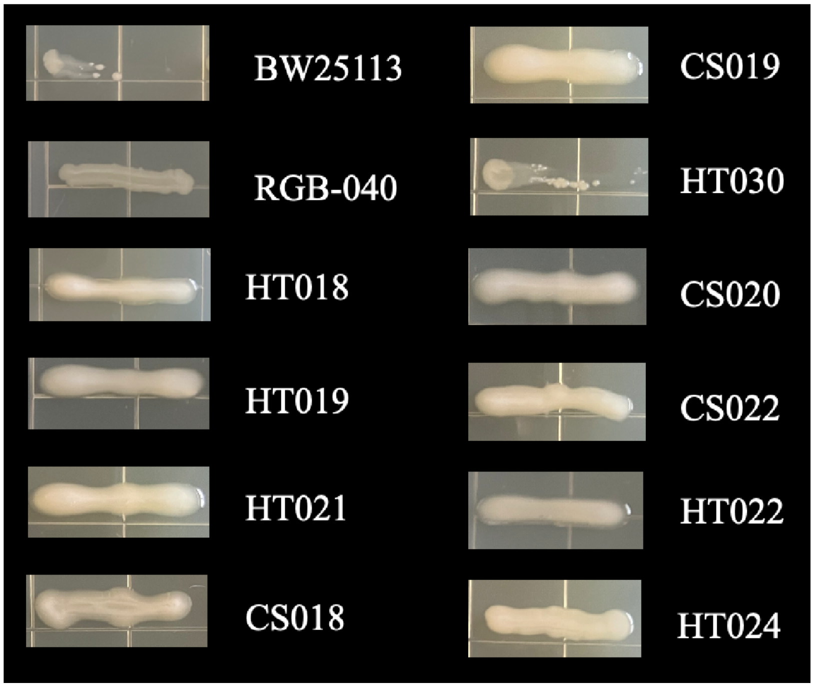

Figure S1. Cross streak results for representative phage resistant mucoid isolates. In each panel, high-titer phage lysate is streaked top to bottom, and bacterial culture is streaked from left to right across the phage. Bacterial growth across the phage line reveals phage resistance (mucoid mutants and *tolC* non-mucoid mutant resistant control RGB-040). Lack of bacterial growth across the phage line reveals sensitivity (wild type). Putative mutant HT030 was non-mucoid and phage sensitive and was omitted from further phenotypic analysis.

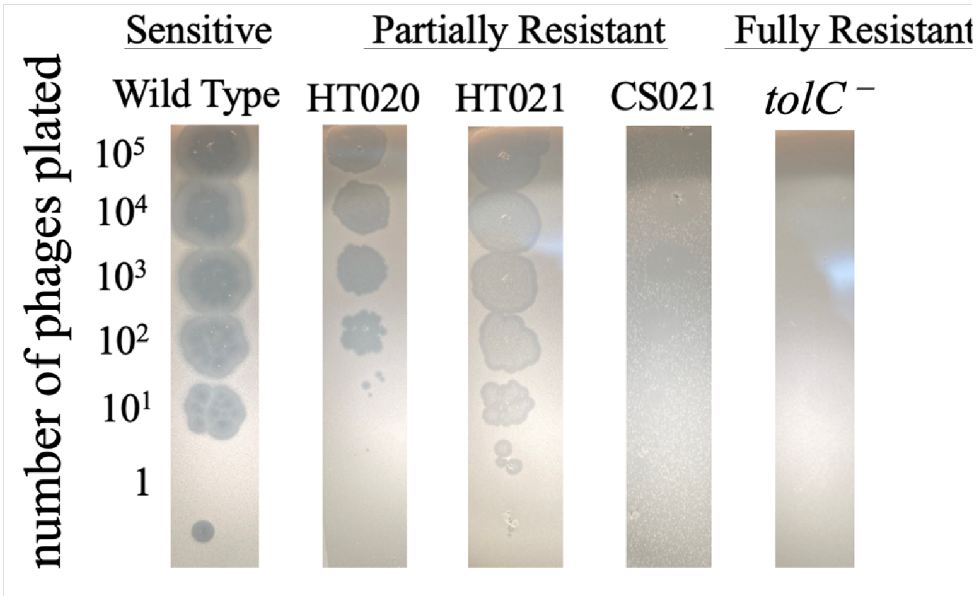

Figure S2. Example EOP assay for mucoid mutants. Phage U136B forms large, clear plaques on the BW25113 wild-type bacterial lawn. Plaquing varies quantitatively (main text Fig. 3) across replicates of the mucoid mutants. When plaques were observed on mucoid mutants, they were varying degrees of hazy, and sometimes barely visible.

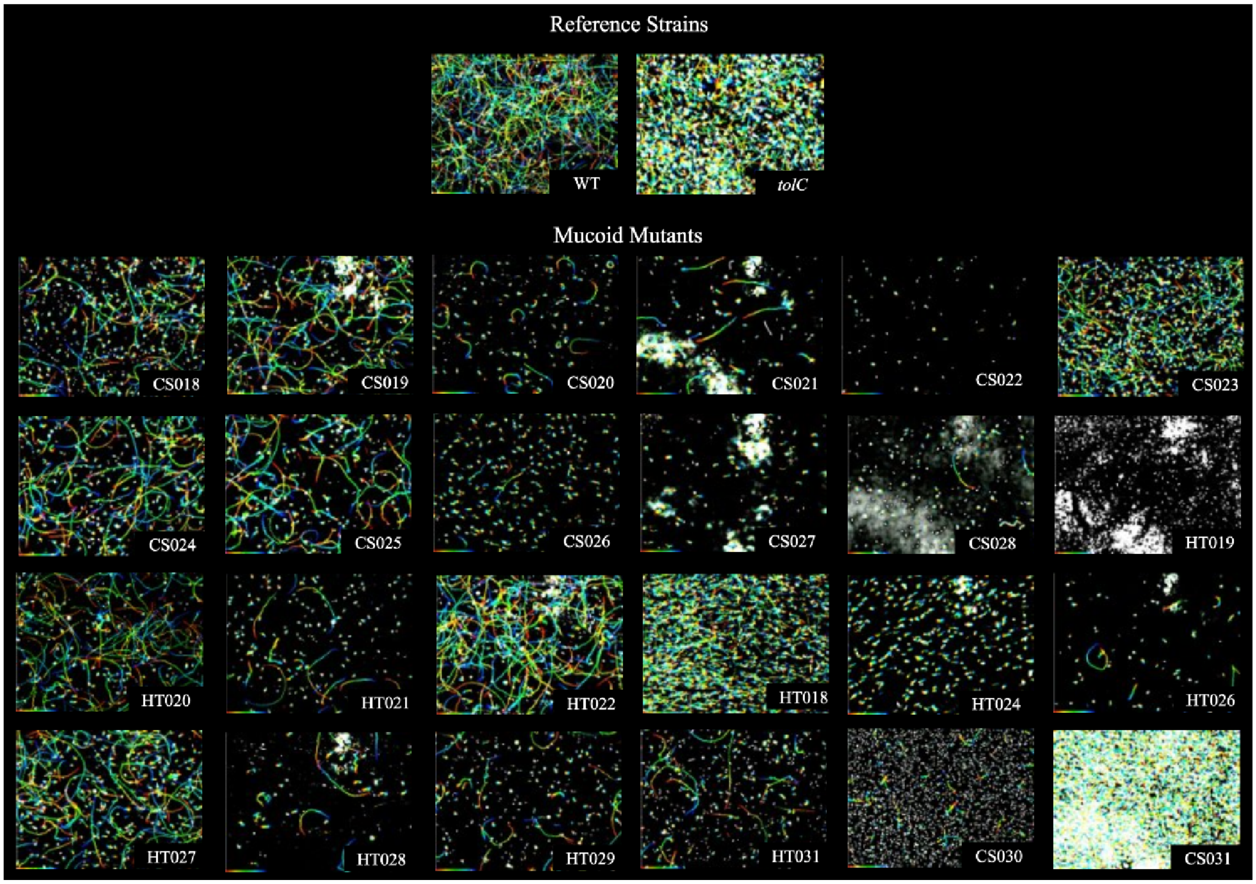

Figure S3. Swimming of wild-type and mutant cells examined by phase contrast microscopy reveals variation in motility among mucoid mutants. For each strain a series of images were taken and colored from red (time 0) to yellow, green, cyan, and finally blue (5 s) and integrated into one image, resulting in "rainbow traces" of swimming cells.

A

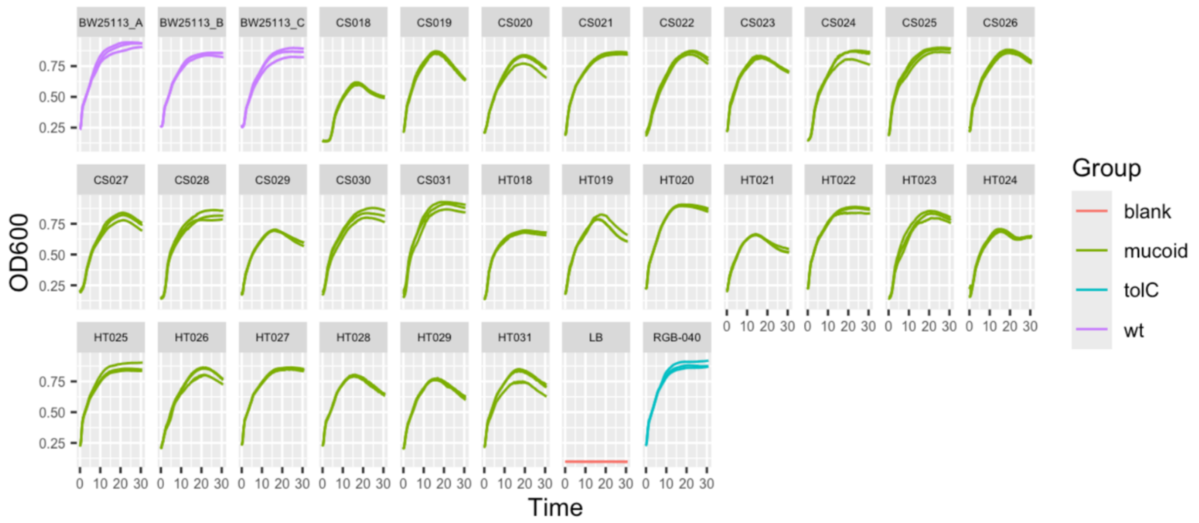

BW25113_A
BW25113_B
BW25113_C
CS018
CS019
CS020
CS021
CS022
CS023
CS024
CS025
CS026
CS027
CS028
CS029
CS030
CS031
HT018
HT019
HT020
HT021
HT022
HT023
HT024
HT025
HT026
HT027
HT028
HT029
HT031
LB
RGB-040
OD600
0.25
0.50
0.75
0 10 20 30
Time
Group
blank
mucoid
tolC
wt

B

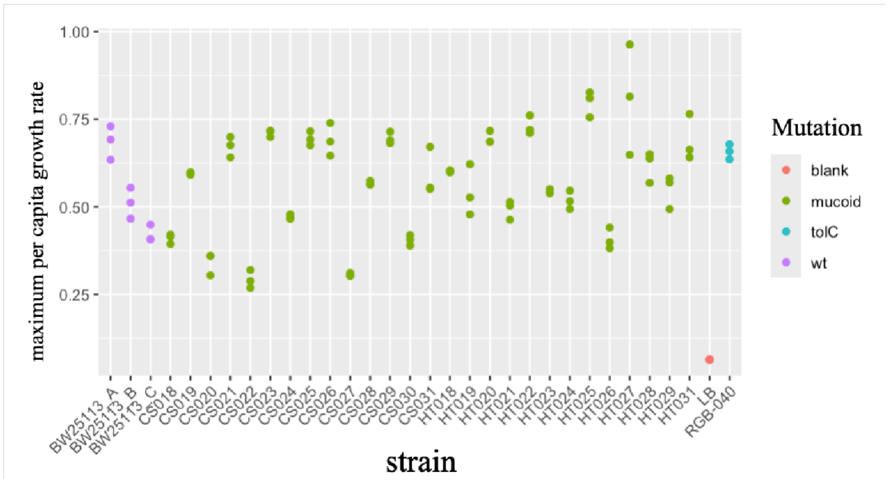

maximum per capita growth rate
0.25
0.50
0.75
1.00
strain
Mutation
blank
mucoid
tolC
wt

C

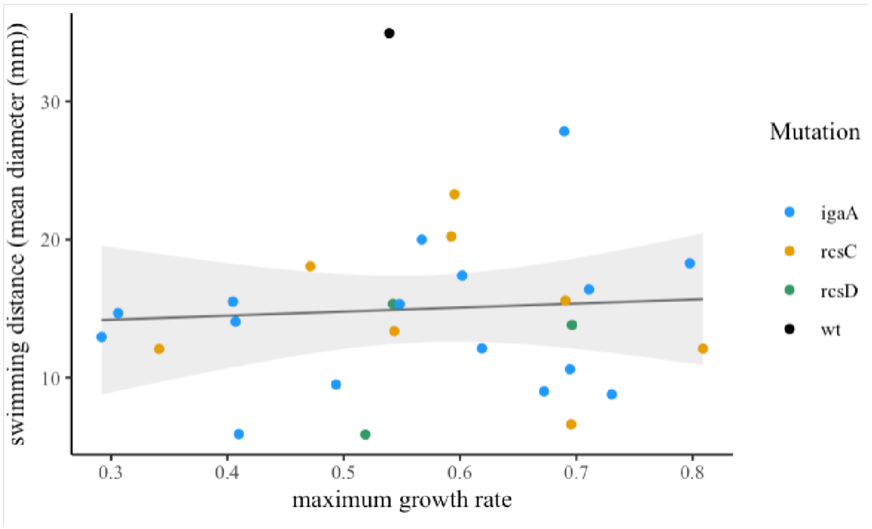

swimming distance (mean diameter (mm))
10
20
30
0.3
0.4
0.5
0.6
0.7
0.8
maximum growth rate
Mutation
igaA
rcsC
rcsD
wt

Figure S4. Analysis of mutant growth parameters. A) Individual growth curves. Lines within each panel represent technical replicates (N = 3). Growth curves were conducted in a 96-well plate reader in LB with orbital shaking and OD_{600} collected every 3 minutes. B) Maximum per capita growth rate calculated from individual growth curves. C) Test of relationship between growth rate and bacterial motility. Individual points represent the 27 mucoid mutants and wildtype. The gray shaded band indicates 95% confidence intervals around the linear model fit (solid black line) including wild type and all mutants ($p = 0.74$, $R^2 = 0.004$).
